# Increasing uptake of hand hygiene education among health care workers via online learning at a Toronto teaching hospital

**DOI:** 10.1186/1753-6561-5-S6-P258

**Published:** 2011-06-29

**Authors:** N Siddiqui, MP Muller

**Affiliations:** 1 St. Mchael’s Hospital, Toronto, Canada; 2 University of Toronto, Toronto, Canada

## Introduction / objectives

Education is a cornerstone of multi-modal strategies aimed to improve hand hygiene compliance among health care workers (HCWs). Major obstacles cited in the literature include limited time availability of HCWs and the reluctance of doctors to attend training sessions. A short, interactive online learning module was developed at St. Michael’s Hospital to update HCWs on best practices related to hand hygiene indications during patient care. Increasing awareness on the transmission and incidence of health care acquired infections, the concept of ‘patient environment’, appropriate glove use as well as hand care were complementary learning points.

## Methods

A formal marketing plan was devised to brand and promote the “quick and interactive” online learning module via: a) the hospital’s intranet home page b) e-mails tailored to staff, physicians and management, c) daily electronic newsletter d) pay stub reminders e) branded promotional T-shirts, f) instructional mini personal hand sanitizers as well as g) raffle prizes.

The uptake of the course was tracked as each HCW was required to log in with their unique ID and password.

## Results

Breakdown of various education session "modes" & uptake among HCWs

Fifteen 30 minute class-room style presentations= approx. 68 HCW (over 4 months)

Thirty 15 minute In-services at in-patient unit nursing stations= approx. 125 HCW (over 6 months)

10 minute Online learning module= 1,484 HCW (over 3 weeks)

## Conclusion

The uptake among HCWs for hand hygiene education at St. Michael's Hospital significantly increased with the introduction and roll-out of an online learning module over a significantly less amount of time.

A formal evaluation is required in order to assess whether knowledge transfer of hand hygiene best practices occured.

## Disclosure of interest

None declared.

